# Health-Improving Effects of Polyphenols on the Human Intestinal Microbiota: A Review

**DOI:** 10.3390/ijms26031335

**Published:** 2025-02-05

**Authors:** Boris V. Nemzer, Fadwa Al-Taher, Diganta Kalita, Alexander Y. Yashin, Yakov I. Yashin

**Affiliations:** 1 Department of Research & Development, VDF FutureCeuticals, Inc., Momence, IL 60954, USA; fadwa.al-taher@futureceuticals.com (F.A.-T.); diganta.kalita@futureceuticals.com (D.K.); 2 Department of Food Science and Human Nutrition, University of Illinois at Urbana-Champaign, Urbana, IL 61801, USA; 3 International Analytical Center of Zelinsky Institute of Organic Chemistry of Russian Academy of Science, Moscow 119991, Russia; yashin@interlab.ru (A.Y.Y.); yashinchrom@mail.ru (Y.I.Y.)

**Keywords:** polyphenols, gut microbiota, fibers, metabolites, bioavailability, nutrition

## Abstract

Dietary polyphenols are garnering attention in the scientific community due to their potential health-beneficial properties and preventative effects against chronic diseases, viz. cardiovascular diseases, diabetes, obesity, and neurodegenerative diseases. Polyphenols are antioxidants that change microbial composition by suppressing pathogenic bacteria and stimulating beneficial bacteria. The interaction of polyphenols with dietary fibers affects their bioaccessibility in the upper and lower parts of the digestive tract. Dietary fibers, polyphenols, their conjugates, and their metabolites modulate microbiome population and diversity. Consuming polyphenol-rich dietary fibers such as pomegranate, cranberry, berries, and tea improves gut health. A complex relationship exists between polyphenol-rich diets and gut microbiota for functioning in human health. In this review, we provide an overview of the interactions of dietary polyphenols, fibers, and gut microbiota, improving the understanding of the functional properties of dietary polyphenols.

## 1. Introduction

Polyphenols are secondary metabolites primarily sourced from fruits, vegetables, cereals, tea, coffee, wine, and beverages. They comprise several classes of phenolic compounds, including flavonoids, phenolic acids, stilbenes, and lignans [1,2]. Polyphenolic compounds possess several health beneficial properties such as anticancer, antioxidant, antimicrobial, and anti-inflammatory activities and play a significant role in the prevention of chronic diseases such as cardiovascular diseases, diabetes, obesity, and neurodegenerative diseases [1,2,3].

Distinctive guidelines and dietary habits among geographically diverse individuals worldwide result in significant variations in polyphenol intake [4,5]. Moreover, polyphenolic content greatly varies with the types of food matrices and their preparation methods [5,6]. In plants, fruits, and vegetables, polyphenols exist in free and bound forms. Most of the analytical, nutritional, and clinical studies of food polyphenols consider phenolic compounds extracted with aqueous organic solvents [7]. These polyphenols are referred to as extractable polyphenols (EPPs). Notably, potentially bioactive polyphenols remain bound in residues comprising macromolecules such as cellulose, protein, and lignin [8]. These polyphenols are referred to as non-extractable polyphenols (NEPPs) and are usually ignored during analysis [8]. Several studies have indicated the presence of abundant NEPPs in specific foods and vegetables such as berries, brown rice, carrots, onions, spinach, apples, and oranges [8,9,10]. Peels, pomace, and seeds also have abundant NEPPs. NEPPs comprise compounds with high-molecular-weight polymers and polyphenols related to cell wall macromolecules (proteins and polysaccharides) or entrapped inside the food matrix [9,10,11]. Major NEPPs constitute condensed tannins, proanthocyanidins, hydrolyzable polyphenols (including hydrolyzable tannins), or polyphenols [8,9,10]. These bioactive compounds exhibit high antioxidant, anti-inflammatory, antidiabetic, and other biological activities [12].

Bioaccessibility is a crucial factor affecting the bioavailability, physiological effects, and biochemical activities of polyphenols. Polyphenols are usually released from food matrices by interacting with digestive enzymes and bacterial microflora in the small and large intestines, respectively, and potentially become bioavailable for absorption [13,14]. Significant differences are observed in the bioavailability of polyphenols, influenced by the type of food matrix, molecular mass, conformations, and physicochemical activity, such as digestibility [14,15]. Consequently, not all phenolic compounds are equally available to exert their functions in the human body after the consumption of phenolic-rich foods. NEPPs, which are not released or absorbed into the gastrointestinal (GI) tract, pass to the lower GI tract, where they are fermented by bacterial flora [10,12]. Upon reaching the lower intestine, polyphenols are biotransformed into their metabolites by gut microbiota and exert various functional and biological effects [11,12,13]. Moreover, these polyphenols and their metabolites modulate the bacterial composition in the colon and influence the gut health of the host [10,11,12]. Several studies have indicated that the interactions of dietary fibers and polyphenols may affect their functional relationship, as well as the integrity and diversity of gut microbiota [9,12,13]. However, there is a complex relationship between polyphenol-rich diets and gut microbiota and their effects on human health, which is yet to be elucidated. In this review, we provide a systematic overview of the effects of dietary polyphenols on the human gut microbiota and their impact on gut integrity and human health. This information will improve the understanding of the function of NEPPs in modulating the gut microbiota composition and reducing health risks.

## 2. Gut Microbiota

The human microbiota comprises 10–100 trillion microorganisms (mostly bacteria, but also viruses, fungi, and protozoa) primarily in the gut [16,17,18,19], similar to the number of human cells [20]. The microbiota abundance gradually increases along the GI tract, with lesser proportion in the stomach and a higher density in the colon (1010–1012/g) and at least 400 bacterial species [19,21]. The GI tract has a large surface area between 250 and 400 m^2^. During a human’s life, an average of 60 tons of food passes through the intestines, along with abundant microorganisms from the environment [22].

A human intestinal microbial gene catalog of 3.3 million genes in the human gut microbiome has been reported, compared with approximately 22,000 genes in the entire human genome [16,19,20,22,23,24]. The microbiota comprises harmless bacteria [16]. Studies have indicated 2172 species in humans, classified into 12 divisions, of which 93.5% belong to phyla *Proteobacteria*, *Firmicutes*, and *Bacteroidetes* [21,25]. In humans, 386 of the identified species are strictly anaerobic and occupy areas such as the oral cavity and the GI tract [25]. Less than 1% of gut bacteria belong to the phyla *Actinobacteria*, *Verrumicrobia*, *Acidobacteria*, or *Fusobacteria*. The small intestine and the colon are dominated by *Bacteroidetes* and *Firmicutes* [21].

Intestinal bacteria provide several benefits in the human intestinal tract. Gut microbes aid in maintaining the intestinal mucus barrier, protecting against pathogens, and developing the immune system. However, these mechanisms may be disrupted by an altered microbial composition, known as dysbiosis, resulting in metabolic diseases such as obesity and type 2 diabetes [26].

Gut bacteria primarily generate energy through fermentation by converting sugars to [16,19,20,22,23,24] short-chain fatty acids (SCFAs) [27]. Gut microbiota synthesizes essential vitamins, including vitamin K, riboflavin, biotin, nicotinic acid, pantothenic acid, pyridoxine, thiamine, B12, and folate. Lactic acid bacteria are significant for vitamin B12 production, which is essential for the normal function of the brain and nervous system and DNA synthesis. *Bifidobacteria* primarily generate folate, a vitamin responsible for human metabolic processes such as DNA synthesis and repair [27,28,29].

The gut microbiota is shaped early in life in the human intestine and undergoes marked changes before reaching equilibrium in adulthood [19,30,31,32,33,34,35]. In the early developmental stages, microbiota is predominantly composed of Actinobacteria and Proteobacteria. Over the age of 65, the microbial community changes and is abundant with Bacteroidetes phyla and Clostridium species [26]. Many factors affect microbiome composition throughout a human’s life. These include microbiome inheritance from the mother, infant delivery mode (vaginal or cesarean), diet, and age-related changes in adults [30]. Everyone has a unique microbiome that develops early in childhood and is stable throughout the lifespan [19,30,31]. The intestinal microbiota reaches a steady state between the ages of 2 and 5 years [19,26]. The gut microbiota is relatively stable in adulthood; however, it exhibits a higher individual variation in older people than in younger adults [36,37,38]. This difference is also affected by genetics, personal hygiene, infection, medication, and diet [30,39,40]. Health in older people is related to gut microbiota modifications. These are associated with changes in the GI tract and diets and a decline in cognitive and immune functions. The gut microbiota’s response to dietary interventions varies among individuals [37,38].

Diet, lifestyle, antibiotics, other drugs, sanitation, and the genetics and immune system of humans may cause changes in microbiota composition [19,30,34,39,40]. Antibiotic treatment significantly disrupts the microbial equilibrium, reducing the abundance and diversity of the community [26]. Dysbiosis occurs when the specific microbial group of the gut microbiota and the abundance and functions of the intestinal microorganisms are altered [19,30,34]. Consequently, illnesses and diseases occur, ranging from cardiovascular, neurological, respiratory, and metabolic illnesses to cancer, inflammatory bowel and skin diseases, Alzheimer’s disease, autism, multiple sclerosis, and allergies [16,19,22,26,30,34]. Similarly, there is an increased risk of metabolic syndromes, including type 2 diabetes mellitus and obesity [34].

Gut microbiota is influenced by geographical locations and differs between developed and developing countries, as well as rural and industrialized regions [33]. Stability is a crucial attribute of good microbial composition. Gut bacteria resist changes induced by ecological stress and return to a balanced state after a stress-related event [34].

Diet accounts for over 50% of the variabilities influencing the microbiota composition over time [34,41,42]. Food availability in developed countries has caused the demand for safer and longer-lasting foods, prompting the industry to produce pre-cooked and ready-to-eat food products containing high amounts of cost-effective, artificial ingredients such as preservatives, colorants, fats, sugar, and salt. The dietary model involving a high consumption of saturated fats and sucrose and a low intake of fiber is called the “Western diet”. A poor diet alters the microbiota and causes disease. Diet markedly influences chronic inflammation, and Western diets are at risk of becoming health hazards that promote high incidences of metabolic diseases and inflammation [34].

## 3. Effect of Diet on Microbiota

Nutrition significantly influences the structure and diversity of the gut microbiota [36,37,43]. Specific foods and diets influence the abundance of gut bacteria and human health [44]. Each individual has a well-defined combination of gut microbes [28]. Differences in the gut microbiota community translate into a distinct ability to process dietary components, resulting in different propensities to diseases [45]. A change in diet exerts different effects on individuals owing to the unique nature of their gut microbiota [41]. Diet amount and content are critical in shaping the microbiota composition and function. Nutrient and microorganism interactions determine beneficial or detrimental effects on health [36].

Dietary additives such as artificial sweeteners and emulsifiers may affect the gut microbiota and modify its composition [36,44]. Artificial sweeteners used as sugar alternatives, which are sweeter than sugars and have fewer calories, may disturb and harm the gut microbiota. Sucralose, aspartame, and saccharin are sugar substitutes that perturb the balance and diversity of the gut microbiota, contributing to metabolic diseases [46]. Similarly, common emulsifiers in processed foods affect the gut microbiota, causing reduced microbial diversity and possibly resulting in metabolic syndromes and inflammatory diseases [47].

Furthermore, food preference causes individual variations in the human microbiota [37]. Obese individuals have more diverse microbial compositions than non-obese individuals do. The proportion of *Bacteroidetes* is lower in obese individuals than in non-obese individuals [43]. Consuming a high-caloric meal with a stable carbohydrate, protein, and fat ratio increases *Firmicutes* and decreases *Bacteroidetes* abundance. This results in reduced diversity of gut microbiota [45,48]. However, a restricted diet with reduced calorie intake increases the diversity [48].

In contrast to other carbohydrates, polysaccharides, such as resistant starch, oligosaccharides, and non-starch polysaccharides, are dietary fibers only accessible to the microbiota of the large intestine; they are not digested in the small intestine [36,43,44,48]. Some dietary fiber sources are fermentable, and bacterial species digest different types of fibers [44]. Gut microbiota, *Bifidobacterium*, *Bacteroides*, *Faecalibacterium*, *Lactobacillus*, and *Roseburia* ferment short-chain fructooligosaccharides to generate SCFAs, primarily butyrate, acetate, and propionate, in the large intestine [44,48]. These SCFAs account for approximately 10% of human energy requirements, leading to reduced calorie needs from the diet [48]. Increased SCFA production correlates with reduced obesity and insulin resistance [44,48]. Abundant fibers in a diet cause a healthy gut microbiome [48].

Some studies showed that the short-term consumption of a diet composed entirely of animal or plant products alters the microbial community and suppresses individual differences in microbial gene expression [45,48,49]. The animal-based diet comprises dairy, meat, and cheeses, while the plant-based diet is rich in grains, legumes, fruits, and vegetables. Fiber consumption is increased through plant diets. Fat and protein intake increases and decreases with animal and plant diets, respectively. The diversity within the sample species remains unchanged under each diet. An animal-based diet has a greater impact on the gut microbiota than a plant-based diet. An animal-based diet increases the number of bile-tolerant microorganisms *Alistipes*, *Bilophila*, and *Bacteroides* but decreases the levels of *Firmicutes* (*Roseburia*, *Eubacterium rectale*, and *Ruminococcus bromii*) that break down plant polysaccharides [48,49]. Both diets have food-borne microbes, including bacteria, fungi, and viruses, that colonize the gut. These imply that the gut microbiome quickly reacts to a modified diet [49]. A diet over 24 h with high fat/low fiber or low fat/high fiber influences the microbiota moderately, with the microbial species being affected differently among individuals. This slight diet modification has certain consequences on human GI health [50].

A “Western diet” prompts significant changes in the microbiota compared with the effect of a low-fat diet and decreases the diversity of the gut microbiota [36,43]. Individuals consuming this diet take in an abundance of animal protein, total and saturated fats, and simple sugars and low quantities of fruits, vegetables, and other plants. Similarly, this diet has low quantities of dietary fiber, non-starch polysaccharides, and resistant starch [45]. It is correlated with a decreased beneficial bacteria abundance of *Bifidobacteria* and *Lactobacilli* and increased organisms belonging to the phyla *Firmicutes* and *Proteobacteria*. This modified microbial community may affect brain function and structure [43]. Its SCFA content is low, and it is associated with a high risk of developing inflammatory bowel disorder, obesity, and type 2 diabetes. This “Western diet” does not benefit humans or the microbiome [43]. Conversely, the “Mediterranean diet”, which is considered the healthier of the two diets, is rich in plant-based proteins. The diet involves the consumption of polyunsaturated fats, fruits, vegetables, bread, olives and olive oil, dairy products, and fish with lower intakes of potatoes, red meat, and sweets [43]. Polyphenol metabolites metabolized by gut bacteria in the colon modulate the gut microbial composition considerably by increasing the abundance of intestinal bacteria like *Bifidobacteria* and *Lactobacilli* and reducing the abundance of *Firmicutes* and *Proteobacteria* organisms. These plant-based diets are correlated with improved cardiovascular health and reduced risk of inflammation. This diet is also associated with enhanced cognitive function [43].

In humans, nutrient digestion and absorption usually occur in the small intestine and stomach. Subsequently, the residual food passes to the colon, where most gut microbes reside [36,43]. Most vitamins are obtained from food and are absorbed in the intestine; only a few are produced by intestinal microorganisms [36]. The microbiome absorbs and produces energy and micronutrients, including essential vitamins, such as vitamin K and many of the water-soluble B vitamins. These are vital for body functions and brain development and cannot be produced by other means [36,43]. Vitamin deficiencies result from perturbed intestinal absorption or inadequate vitamin consumption. Insufficient levels of these vitamins cause cognitive disruption and neurodegenerative deterioration [36]. Moreover, zinc and iron are significant minerals regulated by the microorganisms in the microbiome [43,48]. Iron is essential for oxygen transport and energy production, neurotransmitters, and DNA synthesis. Iron deficiency decreases the abundance of the bacteria that synthesize butyrate in the gut microbiota, resulting in decreased butyrate levels. However, excess iron causes an increased abundance of propionate-producing *Bacteriodaceae* and fewer butyrate-producing *Lachnospiraceae*, leading to increased propionate levels and decreased butyrate levels [43,48]. Zinc is crucial for functions related to gene expression and replication and participates in the regulation and differentiation of neurogenesis [43,48]. Zinc deficiency may be caused by malnutrition, diarrhea, malabsorptive or inflammatory bowel disease (IBD), consumption of antibiotics, metal-chelating agents, or anticonvulsants [48]. This deficiency may alter the gut composition and decrease microbiota diversity and species [43].

The diet of urban industrialized populations has a lower microbial richness than the diet of rural individuals. Bacteroides is predominant in gut microbiomes among individuals in an urban-industrial setting. This diet comprises fiber, fat, protein, polyphenols, and micronutrients, influences the shape of the gut microbiota, and is metabolized by general or specific microorganisms alone or through cross-feeding [36,44,48]. Owing to the decline in dietary fiber intake, microbiota diversity is reduced in individuals of industrialized societies, accompanied by increased food-associated chronic diseases, such as obesity and metabolic syndrome, malnutrition and eating disorders, intestinal inflammatory diseases, or colorectal diseases [51]. Many diseases in industrialized and developing countries are associated with diet. Diet–microbiota interactions have beneficial or detrimental consequences on human health [36].

## 4. Microbiota Correlation with Diseases

The gut microbial population is believed to markedly influence human physiology, considering its contribution to the growth and development of human intestinal cells, supporting its equilibrium [52]. The primary functions of the microbiota are the fermentation of indigestible food components, the synthesis of amino acids, the production of SCFAs, the metabolism of toxins and carcinogens, and the conversion of cholesterol and bile acids [16,27,28,29,39,40]. Gut microbes are critical for humans because they take in energy from food, and the disruption of the gut microbiome may cause obesity or IBD [41,52,53].

Obesity is related to changes in the relative abundances of the two dominant bacterial divisions, the *Bacteroidetes* and *Firmicutes* [54,55]. *Firmicutes* digest long-chain carbohydrates, providing the microbiota with more nutrients, leading to greater energy intake and weight gain [21]. The microbiota of an obese individual readily obtains energy from the diet [54,55].

IBD, a class of inflammatory diseases that include Crohn’s disease and ulcerative colitis, may affect the GI tract due to intestinal dysbiosis. The microbiota of individuals suffering from IBD indicates decreased biodiversity and reduced abundances of *Firmicutes*, *Bacteroidetes*, *Lactobacillus*, *Eubacterium*, and butyrate-producing species. Butyrate is a fatty acid that modulates homeostatic equilibrium within the microbial community and reduces inflammation [56]. While a robust, healthy microbiota protects from dysbiosis-related diseases, such as IBD or metabolic disorders, a resistant, dysbiotic microbiota may cause diseases [57].

The microbiota plays a role in basic biological processes and the development and progression of conditions such as GI cancers and cardiovascular, infectious, liver, metabolic, respiratory, mental or psychological, and autoimmune diseases [41,58,59,60,61,62,63,64,65,66]. The disturbance of the intestinal flora affects the generation of immune mediators and provokes chronic inflammation and metabolic dysfunction [41,63,64,65,66]. Although other diseases may not be directly associated with the microbiota, various mechanisms that include immune, hormonal, and neural pathways may participate in the pathway of diseases such as asthma, food allergies, autism, and major depressive disorder [66,67].

## 5. Interactions of Phenolics, Fibers, and Gut Microbiota

Phenolics and dietary fibers are major concomitant ingredients in plants and food matrices. The association of dietary fibers and polyphenols in food matrices plays a critical role in the bioaccessibility, bioavailability, and biochemical activities of polyphenols [68]. Dietary fibers act as carriers of antioxidants and phytochemicals such as phenolics and deliver these compounds for interaction with the gut microbiota [68]. The composition of polysaccharides and their interaction with polyphenols in food matrices result in the variation in the adsorption and retention properties of polyphenols in the digestion tract [11,68,69]. These interactions could be covalent, non-covalent (hydrogen bonding, ionic bonding, electrostatic interaction, and van der Waals), or hydrophobic [69]. Various factors such as molecular size, polymerization degree in procyanidins, branching degree, surface porosity of polysaccharides, glycosylation percentage, the stereochemistry of flavan-3-ol subunits in proanthocyanidins, gallolylation percentage, and steric hindrance affect the interactions between polyphenols and polysaccharides [70,71,72,73,74]. Simple phenolic acids such as ferulic acid, p-hydroxyphenyl, syringyl, and p-coumaroyl acids covalently associate with plant cell wall arabinogalactans and arabinoxylanes in sugar beet and wheat, bamboo, maize, and spinach. Proanthocyanidins bind to the cell wall via non-covalent interactions like hydrogen bonding and van der Waals forces [12]. Flavonols such as rutin, isoquercetin, and quercitrin were reportedly parts of NEPPs in fruits or leaves, tomato peel, and tropical and subtropical fruits. However, the exact interaction of flavonoids and the cell wall is partially understood. Hu et al. (2023) reported that specific non-covalent interactions occur in pectin-polyphenols via hydrophobic interactions and hydrogen bonding [75]. Watrelot et al. (2013) reported that aromatic groups in polymeric procyanidin led to a hydrophobic interaction with pectin polysaccharides [76]. Fernandes et al. (2020) reported that branched arabinan-pectic polysaccharides hinder the potential interactions between polyphenols and polysaccharides, where linear arabinans exhibited 2–8-fold higher retention for phenolic compounds such as phloridzin and chlorogenic acid than the branched arabinans [77].

Polyphenol–polysaccharide interaction significantly influences polyphenol extractability and potential nutritional biochemical properties [78,79,80]. While most polyphenols are extracted using a conventional water–organic solvent solution, the optimum extractability depends on the polyphenol–polysaccharide ratio, pH, ionic strength of the extracting solvents including the time, temperature, and atmospheric conditions [9,10,13]. As NEPPs are extracted through acidic, alkaline, or enzymatic hydrolysis, these factors also influence the extraction rate of bound phenolics from the food matrix [79].

**Figure 1:**
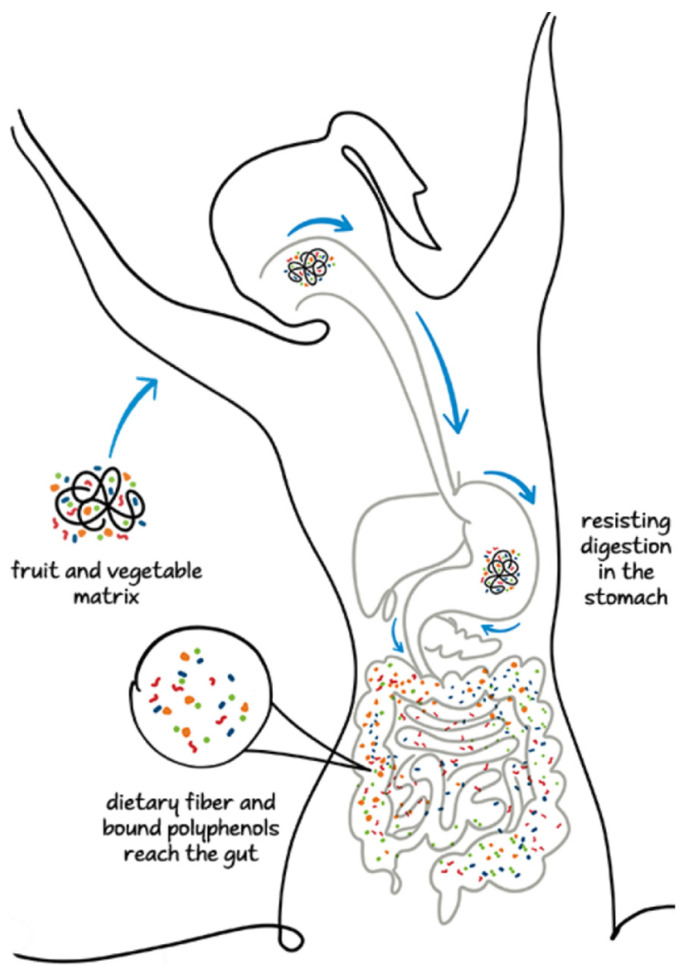
Fate of inaccessibility of polyphenols from food matrices in digestive system.

The polyphenol–polysaccharide interaction properties strongly influence the rate of polyphenol release at various stages of the digestive system involving the upper and lower GI tracts and colon [68]. A fraction of polyphenols is partially released in the small intestine owing to the weakening of the ester-phenolic carbohydrate bonds and the perturbation of phenolic polysaccharide interactions. An abundant fraction of NEPPs reaches the lower intestinal tract, where it undergoes fermentation by the microflora in the colon and produces diverse metabolites, including phenolic acids such as ferulic acid, valeric acid, SCFA, and phenolic-SCFA conjugates [68,80,81]. In the colon, some hydrolytic enzymes, including carbohydrolases and protease, are secreted by microbiomes such as *Bifidobacterium* spp., *Clostridium* spp., and *Lactobacillus* spp. and weaken the phenolic–polysaccharide interaction by hydrolyzing the covalent bonds between phenols and carbohydrates and the glycosidic linkages in polyphenol compounds [68,79]. In some cases, the metabolites are biologically more active than the native compounds [68]. Figure 1 represents the fates of the bioaccessibility of free and bound phenolic compounds during the entire digestive process.

In a preclinical study, ferulic acid-bound wheat bran produced more bioavailable phenolic compounds than did the free ferulic acid extract alone [82]. Vitaglione et al. (2015) found that whole grain fiber enriched with polyphenols acted as a carrier of bioactive phenolic compounds to the lower gut, while bound phenolics were not bioaccessible in the small intestine, making them bioavailable for the colon [83].

Sugarcane contains fiber-bound phenolics such as phenolic acids (ferulic acid, coumaric acid, hydroxybenzoic acid, vanillic acid, syringic acid), flavones (apigenin, luteolin, and tricin), flavanone (5,7-dihydroxyflavanone), flavanol (catechin), and flavonol (6,8-dihydroxykaempferol) [84,85]. The fibers in sugarcane act as carriers of these phenolic and polyphenolic compounds via the small intestine to the colon for fermentation [84,85]. Apple fiber contains significant amounts of non-extractable polyphenolic compounds that originate from the interactions of dietary fiber and polyphenols and affect gut microbial diversity [86]. The procyanidin–polysaccharide interactions within the apple matrices decrease procyanidin degradation by the human gut microbiota [87].

In addition to the beneficial effects as carriers of phenolics to the colon, fibers and polyphenols act synergistically with potential biochemical properties. Previous studies have reported that the individual physiological effects of NEPPs or dietary fibers are less pronounced than the combined effects of both. They provide a synergistic effect, with enhanced fermentation of NEPPs and bioavailability of metabolites for their various physiological functions. During the entire fermentation process, fibers or polysaccharides could act as prebiotics for colonic microflora. For example, Saura-Calixto et al. (2010) demonstrated that in vitro fermentation with dietary fiber-enriched NEPPs and fiber alone showed 53% and 23% fermentability, respectively [80]. The association of a fiber–polyphenol matrix could affect the microbiota community. In a randomized control study, Vitaglione et al. (2015) found that whole wheat grain intake increased *Bacteroidetes* and *Firmicutes* proportions but decreased *Clostridium* abundance, accompanied by reduced inflammatory markers, while refined wheat grain lowered *Bifidobacteriales* abundance and increased *Bacteroidetes* abundance [83].

A reciprocal relationship between polyphenols and the gut microbial community could occur during the digestion processes of food matrices. After reaching the polyphenols to the colon, the gut microbiota transforms polyphenols into varieties of bioactive metabolites, and then those metabolites influence the diversity of the microbiome, including the Firmicutes/Bacteroidetes F/B ratio. In most cases, phytochemicals are transformed into low-molecular-weight metabolites [88,89]. Most food polyphenolic compounds are present as monosaccharides, organic acid, and their conjugates. During the digestive process, these derivatives undergo microbial enzymatic catabolism via O-deglycosylations and ester hydrolysis, C-ring cleavage, delactonization, demethylation, dehydroxylation, and the reduction of double-bond compounds [89]. Various microbial enzymes transform polymeric NEPPs into low-molecular-weight metabolites [90]. During this process, they are further depolymerized and catabolized through microbial enzyme-induced delactonization and decarboxylation and finally form phenolic acids such as phenylacetic acid, phenyl propionic acid, phenylvaleric acid, cinnamic acid, benzoic acid, and hippuric acid [91,92]. Finally, these metabolites are delivered to the liver after passing through colonocytes, followed by systemic circulation and subsequent absorption by different tissues or elimination through the urine [93]. During the microbial transformation of hydrolyzable tannins, the gallotannins are hydrolyzed to gallic acid and glucose and converted to pyrogallol and phloroglucinol and, subsequently, to their final products such as acetate and butyrate. Ellagitannins are biotransformed by the cleavage of the lactone ring, decarboxylation, and dehydroxylation [89].

Furthermore, polyphenols and gut microbiota are interrelated. The effects of phenolic compounds on the abundance and diversity of gut microbiota have been studied extensively. The incubation of flavanols such as catechin and epicatechin from tea, with human fecal bacteria reduced the growth of certain pathogens such as *Clostridium difficile*, *Clostridium perfringens*, *Streptococcus pyogenes*, and *Streptococcus pneumoniae* [94]. Another study by Tzounis et al. (2008) on the batch-culture incubation of human fecal bacteria with (+)-catechin observed that catechin exerted a strong prebiotic effect by enhancing the growth of the *Erec* group, *Bifidobacterium* spp., and *E. coli*. significantly [95]. Catechin also inhibited pathogenic *Clostridium histolyticum* growth. The growth of *Lactobacillus* spp., *Bifidobacterium* spp., and butyrate-producing *Erec* were enhanced in a batch-culture fermentation with anthocyanins such as malvidin-3-glucoside [96]. Moreover, when a mixture of anthocyanins with malvidin-3-glucoside, delphinidin-3-glucoside, petunidin-3-glucoside, peonidin-3-glucoside, and cyanidin-3-glucoside was fermented, an increase in the abundances of *Lactobacillus* spp. and *Bifidobacterium* spp. occurred compared with malvidin-3-glucoside alone [96]. In vitro (batch fermentation of human fecal bacteria) and in vivo (rat) studies of quercetin and rutin demonstrated that they enhanced the growth of *Bifidobacterium* spp. (at 10 ug/mL) and reduced the Firmicutes/Bacteroidetes F/B ratio [97,98]. Naringenin inhibited the growth of *E. coli*, *S. aureus*, *S. typhimurium*, *L. rhamnosus*, *B. galactorunicus*, *Lactobacillus* sp., *E. caccae*, *B. catenulatum*, and *R. gauvreauii*. Isoflavones such as daidzein and genistein showed antibacterial activities against *E. coli*, *S. typhimurium*, and *S. rhamnosus* [99]. Phenolic acids such as hydroxycinnamic acid inhibited the growth of *E. coli*, *S. aureus*, *S. typhimurium*, and *L. rhamnosus*. Caffeic acid and chlorogenic acid reduced the Firmicutes/Bacteroidetes F/B ratio of the fecal bacterial community [98]. Gallic and caffeic acids inhibited the growth of pathogenic bacteria *C. perfringens*, *C. difficile*, *Listeria monocytogenes*, and *S. aureus* [99].

## 6. Interactions of Phenol-Rich Sources and Gut Microbiota

### 6.1. Pomegranate

Pomegranate is a rich phenol source, comprising extractable and non-extractable phenolic compounds. Pomegranate has abundant antioxidants and anti-inflammatory bioactive compounds, primarily ellagitannins and anthocyanins, which are concentrated in the peel, membranes, and piths [100]. Sun et al. (2021) identified ten NEPPs in pomegranate peel, among which they separated six [101]. These were recognized as β-sitosterol-3-O-glycoside, β-sitosterol, ursolic acid, corosolic acid, asiatic acid, and arjunolic acid. Ursolic and asiatic acids demonstrated antimicrobial activity against various pathogens [101]. Pomegranate peel extract hindered the growth of *C. perfringens*, *C. ramosum*, *S. aureus*, and *C. clostridioforme* and markedly increased *Bifidobacterium* breve and *Bifidobacterium* infantis [102]. Pomegranate by-product increased the growth of total bacteria, *Bifidobacterium* spp., and *Lactobacillus–Enterococcus* and SCFA production [103]. This implies that consuming pomegranate peel extract improves health through the modulation of gut microbiota [100].

Pomegranate juice is considered a significant source of phenolic compounds, with anthocyanins being the most crucial [104], along with lignans [105], gallagyl-type tannins, ellagic acid derivatives, and other hydrolyzable tannins, which enhance the antioxidant activity of the juice [106,107]. Pomegranate juice is superior to other juices, as it is enriched with polyphenols and has a higher antioxidant activity than do common juices or extracts [108]. The antioxidant activity of pomegranate juice reported by Gil et al. (2000) was thrice higher than that of red wine and green tea [106]. Similarly, pomegranate juice has displayed notable anti-atherosclerotic (prevention of oxidative stress and inflammation in the arterial wall), anti-hypertensive, and anti-inflammatory effects in humans. The gut microbiota composition is not significantly altered by the consumption of pomegranate juice [109].

### 6.2. Berries

Berries contain an abundance of polyphenolic compounds, such as phenolic acids, flavonols, and anthocyanins. Their polyphenol profile varies from one species to another. Most of the berry polyphenols reach the gut and metabolize smaller phenolic compounds there through the action of microorganisms [110]. Polyphenols and their metabolites can also modulate microbial populations, which can lead to an increase in the healthy bacteria *Bifidobacterium*, *Lactobacillus*, and *Akkermansia*. The consumption of berries has led to beneficial effects, primarily anti-inflammatory activity and the promotion of apoptosis [110].

Cranberries are among the richest sources of polyphenols, comprising flavonols, particularly quercetin, anthocyanins, and oligomeric A-type proanthocyanidins [111]. Cranberry polyphenols (CPs) have several biological effects, such as antioxidant, antibacterial, antifungal, urinary tract protection, cardioprotective, and anticancer activities [111]. Certain CPs, particularly proanthocyanidins, are poorly absorbed in the small intestine. These polyphenols, therefore, are bioaccessible only in the large intestine, where they may interact with the microbiota and modify gut microbial composition to maintain the metabolic health of humans [112]. A cranberry-rich diet may be beneficial in preventing metabolic syndromes associated with chronic diseases and improving obesity-induced dysbiosis. The consumption of cranberry extracts rich in polyphenols, such as proanthocyanidin polymers, promotes the growth of bacteria, such as *Barnesiella*, *Akkermansia muciniphila*, *Lactobacillus*, and *Coriobacteriales*, while repressing the growth of others, such as *Oscillibacter*, *Romboutsia*, *Ruminiclostridium*, and *Roseburia*. CPs could inhibit nonbeneficial bacteria in the human microbiota and facilitate the growth of probiotic bacteria [112]. Cranberries comprise anthocyanins, the major polyphenols in the EPP fractions, while phenolic acids are abundant in the NEPP fraction [113]. Han et al. (2019) showed that the oxygen radical absorbance capacity of the NEPPs is significantly higher than that of the EPPs. The EPP and NEPP fractions from cranberries are bioactive, and the NEPPs showed potentially stronger anti-inflammatory and anti-colon cancer activity than the EPs [113].

The most important polyphenols found in blueberries are anthocyanins, and the content varies depending on the blueberry variety. For example, Giacalone et al. (2015) observed a wild type of blueberries contained 340 mg/100 g of fresh fruit, which is more than 50% of the total polyphenol content in the pulp and skin of the blueberries. Polyphenol content increases as the fresh fruit matures [114]. The primary anthocyanins contained in blueberry are pelargonidin-3-D-galactoside, delphinidin-3-glucoside, and malvin-3-O-glucose. Pelargonidin-3-D-galactoside may prevent and control illnesses associated with high cholesterol. Delphinidin-3-glucoside can prevent the spread of breast and colon cancer [115]. The consumption of blueberries, which are rich in anthocyanins, increases antioxidant activity. Blueberry polyphenols are good neuroprotective agents that can decelerate aging and treat neurological disorders [114]. Moreover, blueberry polyphenols regulate the composition of gut microbiota. The diversity of gut microbes improves with an increasing amount of *Akkermansia* and a decreased amount of *Proteobacteria*, suggesting that blueberries may correct gut dysbiosis. The consumption of blueberries increases the abundance of *Bifidobacterium*, *Faecalibaculum*, *Lactobacillus*, *Parabacteroides*, and *Roseburia* organisms. *Bifidobacterium* and *Lactobacillus* have beneficial effects such as reduction in low-grade inflammation and better gut barrier function [116]. The other three bacteria produce SCFAs with *Roseburia*, especially yielding butyrate. Gut microbiota ferment the fiber in blueberries, which can change the activity of bacteria responsible for the breakdown of polyphenols. Moreover, the metabolism of polyphenols can be reduced by binding fibers with proanthocyanidins, decreasing the production of bioactive anti-inflammatory metabolites [116].

Elderberry is abundant in bioactive compounds, such as polyphenolic compounds and terpenoid compounds. The polyphenolic compounds include phenolic acids, flavanones, flavonols, and anthocyanins in elderberry leaves and fruits [117]. Liu et al. (2022) determined that the primary phenolic acids in elderberry fruit were gallic acid and gentisic acid, while the main flavonoids were rutin and quercetin [117]. The primary phenolic acids in elderberry flower extracts were chlorogenic acid, 5-p-coumaroylquinic acid, and dicaffeoylquinic acid, whereas rutin and naringenin were the main flavonols and flavanones detected in the extracts. The major anthocyanins in elderberry fruits were cyanidin 3-O-sambubioside and cyanidin 3-O-glucoside. Additionally, 13 polyphenolic compounds, mainly anthocyanins, were detected in both elderberry fruits and branches [117]. Overall, the elderberry flowers are more abundant in polyphenolic compounds than the fruits. Moreover, the number of polyphenols was higher in fruits and flowers than in the other parts of the elderberry plant, its branches, leaves, and floral stems. The primary triterpenoids detected in elderberry fruits were ursolic and oleanolic acids [117]. Elderberry fruits exhibit various health benefits such as antioxidant, anti-inflammatory, anticancer, anti-influenza, antimicrobial, antidiabetic, cardiovascular-protective, and neuroprotective functions [117]. Teets et al. (2024) observed that the consumption of elderberry juice significantly increased the abundance of *Firmicutes* and *Actinobacteria* and decreased the *Bacteroidetes* phyla [118]. Drinking elderberry juice increased *Faecalibacterium*, *Ruminococcaceae*, and *Bifidobacterium* and decreased *Bacteroides* and lactic acid-producing bacteria [118].

### 6.3. Tea

Tea (from the plant *Camellia sinensis*) contains substantial flavonoids, with catechins being the major class, including epicatechin, epigallocatechin, epicatechin-3-gallate, and epigallocatechin-3-gallate. Additionally, tea contains flavanols, such as quercetin, kaempferol, myricetin, and their glycosides [100]. The NEPP content in green tea is four times lower than that of EPP. The antioxidant activity of NEPPs is lower than that of EPPs but higher than that of vitamin C. NEPPs also display an α-glucosidase inhibitory effect. Gallocatechin, epigallocatechin, catechin, epicatechin, and caffeine were found to be the most abundant compounds in green tea [119]. Phenolic compounds in green tea reduce the abundance of C. perfringens and other Clostridium species [100]. A study of 10 participants showed that the proportion of *Bifidobacteria* increased after drinking green tea and decreased after stopping tea consumption. However, the makeup of the *Bifidobacterium* species showed no significant changes in each sample. This indicates that green tea may act as a prebiotic and enhance the colon microbiota by increasing the proportion of the *Bifidobacterium* species [120].

### 6.4. Cocoa

Cocoa and its products are enriched with polyphenols, such as flavan-3-ols, in the form of epicatechin and catechin, as well as type-B proanthocyanidins [98,121]. These polyphenols may help alleviate conditions such as hypertension, oxidative stress, cancer, atherosclerosis, diabetes, and diverse central nervous system disorders related to gut microbiota [100]. Many cocoa polyphenols reach the colon, where they are degraded into smaller metabolites by gut microbiota. Cocoa metabolites improve gut health by exerting anti-inflammatory and antioxidant effects, positively affecting immunity and reducing the risk of various diseases [121]. Cocoa polyphenols exert prebiotic effects, as they promote the growth of beneficial bacteria, such as *Lactobacillus* and *Bifidobacterium*, but reduce the abundance of harmful bacteria, such as certain *Clostridium* species [121]. Cocoa intake significantly reduces the abundance of the *Bacteroides*, *Staphylococcus*, and *Clostridium* genera. *Lactobacillus* spp. and *Bifidobacterium* spp. abundances were increased significantly with the intake of high-cocoa flavanol, while the *C. histolyticum* group, including the *C. perfringens* pathogen, which contributes to human disease, showed a decreased abundance [100].

### 6.5. Red Wine

Wine has abundant phenolic compounds, comprising a mixture of flavonoids (flavan-3-ols and anthocyanins), non-flavonoids (resveratrol, cinnamates, and gallic acid), oligomeric and polymeric proanthocyanins, catechins, and phenolic acid [122,123]. Dolara et al. (2005) reported that the wine polyphenolic extract contained 4.4% anthocyanins, 0.8% flavanols, 2.0% phenolic acids, 1.4% catechin, 1.0% epicatechin, and 28% proanthocyanidin [124]. Drinking red wine is associated with multiple health benefits, ranging from a reduction in cardiovascular disease risk factors, metabolic syndrome, and depression to improved cognition and positive effects related to gut microbiota diversity [123,124,125,126,127]. Polyphenols reduce oxidative stress, exhibit anti-inflammatory activity, and promote beneficial gut bacteria [124]. Polyphenol-treated rats showed higher *Bacteroides*, *Lactobacillus*, and *Bifidobacterium* spp. proportions than did the controls. However, *Bacteroides*, *Clostridium*, and *Propionibacterium* spp. showed higher abundances in the control rat feces. These imply that wine polyphenols simulate the favorable effects of fibers and prebiotics on the colon bacteria [124]. Queipo-Ortuno et al. (2012) detected an increase in four major bacteria phyla (*Proteobacteria*, *Fusobacteria*, *Firmicutes*, and *Bacteroidetes*) and significant increases in *Bifidobacterium* and *Prevotella* abundance after drinking red wine. No changes were observed in the *Actinobacteria* phyla; however, significant decreases were detected in the *Clostridium* genera and *Clostridium histolyticum* group following red wine consumption [127]. This was associated with decreased blood pressure, triglycerides, total cholesterol, and C-reactive protein [125,126,127].

### 6.6. Grapes

Grapes (Vitis vinifera) are rich in bioactive compounds, particularly polyphenols, which primarily comprise proanthocyanidins, anthocyanins, flavonols, phenolic acids, and stilbenes [128,129]. The polyphenol contents depend on the grape parts. Most of the polyphenols, primarily proanthocyanidins, are in the grape seed [130,131]. The major flavonoids in grapes include flavan-3-ols, flavonols, and anthocyanins, whereas non-flavonoids primarily comprise phenolic acids and stilbenes. Flavan-3-ols are the richest flavonoids in grapes, stored primarily in the seeds but are rarely detected in the skin [132]. Grape skin contains relatively substantial amounts of anthocyanins, whereas almost no anthocyanins are in grape seed [132,133]. Grape polyphenols have diverse health benefits, such as antioxidant, anti-inflammatory, gut-microbiota-modulating, anti-obesity, cardioprotective, hepatoprotective, antidiabetic, and anticancer effects [134]. Han et al. (2020) reported that grape extract restored the dysbiosis of gut microbiota produced by a high-fat diet by increasing the B/F ratio and the abundances of *Bifidobacteria*, *Clostridia*, and *Akkermansia* [135].

Table 1 presents the polyphenol dietary sources, their human health benefits, and beneficial bacteria, where available in the literature. Polyphenols reduce the concentration of pathogenic bacteria and increase the population of those that are beneficial. Consequently, polyphenols change the microbiota composition to a more beneficial profile, favoring human health.

## 7. Conclusions

This review summarizes the basic relationship between fibers, polyphenols, and gut microbiota and their modulatory role on human gut health. Also, this review offers fundamental insights for researchers, clinicians, and food scientists aiming to harness nutrition to support gut microbiome diversity and health. Gut microbiota influences the biotransformation of polyphenols to bioactive metabolites that control the diversity and quality of microbiomes. Fiber–polyphenol interaction significantly influences the efficient release of polyphenols at various stages of the digestive process and their bioaccessibility in the colon, where they are fermented by microflora to produce many metabolites with enhanced bioavailability and bioactivities. Moreover, the composition and structure of dietary fibers affect the interaction level and influence the diversity and activity of the gut microbiota. A complex interactive relationship exists between food matrices containing fibers and polyphenols and their role in modulating the gut microbiota. The formulation of new products with the desired fibers and polyphenols is promising in improving human gut health by modulating the microbiota. Future studies exploring a) how the types of dietary fibers and polyphenols influence microbial diversity and metabolite production and b) the optimal combinations of dietary fibers and polyphenols that maximize bioaccessibility and bioactivities in the colon would be valuable for developing cost-effective and consumer-friendly new functional food products.

## Figures and Tables

**Table 1:** Polyphenol dietary sources, their human health benefits, and beneficial bacteria.

Food Products	Polyphenols	Health Benefits	Beneficial Bacteria	References
***Pomegranate***	*β*-sitosterol-3-O-glycoside, ursolic acid, corosolic acid, asiatic acid, arjunolic acid, and ellagitannins and anthocyanins	Antimicrobial activity		[101]
	-	Antioxidant and anti-inflammatory effects	*Bifidobacterium breve* and *Bifidobacterium infantis*.	[102]
	-	-	*Bifidobacterium* spp. and *Lactobacillus-Enterococcus*.	[103]
***Cranberries***	Flavonols, anthocyanins, and oligomeric A-type proanthocyanidins	Urinary tract protection; antioxidant, antibacterial, and antifungal effects; and cardioprotective and anticancer activities	-	[111]
	-	Prevents metabolic syndromes associated with chronic diseases and improves obesity-induced dysbiosis.	*Barnesiella*, *Akkermansia muciniphila*, *Lactobacillus*, and *Coriobacteriales.*	[112]
***Blueberries***	Major anthocyanins (pelargonidin-3-D-galactoside, delphinidin-3-glucoside, and malvin-3-O-glucose)	May prevent and control illnesses associated with high cholesterol; Can prevent the spread of breast and colon cancer.	-	[115]
	-	Antioxidant activity increases after consumption of blueberries. Good neuroprotective agents that can decelerate aging and treat neurological disorders.	-	[114]
	-	-	Increased amount of *Akkermansia* and a decreased amount of *Proteobacteria*. Increased abundance of *Bifidobacterium*, *Faecalibaculum*, *Lactobacillus*, *Parabacteroides*, and *Roseburia* organisms.	[116]
***Elderberries***	Phenolic acids, flavanones, flavonols, and anthocyanins; ursolic and oleanolic acids	Antioxidant, anti-inflammatory, anticancer, anti-influenza, antimicrobial, antidiabetic, cardiovascular-protective, and neuroprotective functions	-	[117]
			Increased abundance of Firmicutes and Actinobacteria and decreased the Bacteroidetes phyla; Increased *Faecalibacterium*, *Ruminococcaceae*, and *Bifidobacterium* and decreased *Bacteroides* and lactic acid-producing bacteria.	[118]
***Green tea***	Flavonoids (catechins, the major class includes epicatechin, epigallocatechin, epicatechin-3-gallate, and epigallocatechin-3-gallate), flavanols, (quercetin kaempferol, myricetin, and their glycosides)	Antimicrobial effect	-	[100]
	Gallocatechin, epigallocatechin, catechin, epicatechin, and caffeine	Antioxidant and α-glucosidase inhibitory effects	-	[120]
	-	-	Increased the proportion of the *Bifidobacterium* species.	[121]
***Cocoa***	Flavan-3-ols, in the form of epicatechin and catechin, as well as type-B proanthocyanidins	-	-	[100,121]
	-	Alleviate hypertension, oxidative stress, cancer, atherosclerosis, diabetes, and diverse central nervous system disorders Weight loss and reductions in C-reactive protein	Decreased concentrations of *Bacteroides*, *Staphylococcus*, and *Clostridium* genera Increased concentrations of *Lactobacillus* spp. and *Bifidobacterium* spp. Decreased abundance of *C. histolyticum* group, a group that includes elligatannins *Clostridium perfringens* pathogen and contributes to human disease.	[100]
	-	Improve gut health, possess anti-inflammatory and antioxidant effects, positively affecting immunity, and reduce the risk of various diseases	Promotes the growth of beneficial bacteria such as *Lactobacillus* and *Bifidobacterium* Reduced abundance of *Clostridium* genus	[121]
***Red wine***	Flavonoids (flavan-3-ols and anthocyanins), non-flavonoids (resveratrol, cinnamates, and gallic acid), oligomeric and polymeric proanthocyanins, catechins, and phenolic acid	-	-	[122,123]
	4.4% anthocyanins, 0.8% flavanols, 2.0% phenolic acids, 1.4% catechin, 1.0% epicatechin, and 28% proanthocyanidin	-	-	[124]
	-	Reduction in cardiovascular disease risk factors, metabolic syndrome and depression, improved cognition, and health benefits associated with gut microbiota diversity	-	[122,123,124,125]
	-	Reduced oxidative stress. Anti-inflammatory activity	Increased abundance of *Bacteroides*, *Lactobacillus*, and *Bifidobacterium* spp.	[123]
	-	Decreased blood pressure, triglycerides, total cholesterol, and C-reactive protein	Increased abundance of four major bacteria phyla (*Proteobacteria*, *Fusobacteria*, *Firmicutes*, and *Bacteroidetes*) and the *Bifidobacterium* and *Prevotella* species Decreased abundance of *Clostridium* genus and *Clostridium histolyticum* group.	[127]
***Grapes***	Proanthocyanidins, anthocyanins, flavonols, phenolic acids, and stilbenes	-	-	[128,129]
	Major flavonoids (flavan-3-ols, flavonols, and anthocyanins) and non-flavonoids (phenolic acids and stilbenes)	Antioxidant, anti-inflammatory, anti-obesity, cardioprotective, hepatoprotective, antidiabetic, and anti-cancer effects	-	[134]
	-	-	Increased *Firmicutes*/*Bacteroidetes* ratio and the abundances of the *Bifidobacteria*, *Clostridia*, and *Akkermansia* genera	[135]
